# Analysis of Genetic Diversity and Population Structure in Three Forest Musk Deer Captive Populations with Different Origins

**DOI:** 10.1534/g3.119.400001

**Published:** 2019-02-08

**Authors:** Jiamin Fan, Xueli Zheng, Hongyong Wang, Hong Qi, Benmo Jiang, Meiping Qiao, Jianwen Zhou, Shuhai Bu

**Affiliations:** * College of Life Sciences, Northwest A&F University, Yangling 712100, Shaanxi, China; † College of Forestry, Northwest A&F University, Yangling 712100, Shaanxi, China; ‡ Fengxian Feng Chun Ji Min Breeding Limited Company, Baoji 721702, Shaanxi, China; § Qingchuan Jiu Yao Musk Deer Development Limited Company, Guangyuan 628109, Sichuan, China; ** Ecological and Wildlife Conservation Management Station in Feng County, Baoji 721700, Shaanxi, China

**Keywords:** Forest musk deer, Genetic variation, Population structure, Musk secretion, RAD sequence

## Abstract

Musk deer (Moschidae), whose secretion is an expensive and irreplaceable component of traditional medicine, have become endangered in the wild due to habitat fragmentation and over-exploitation. In recent years, China has had success in the artificial breeding of forest musk deer, thus relieving the pressure on wild populations. However, many farmed populations are experiencing degradation, and little genetic information is available for conservation management. In this study, we selected 274 individuals from three typical captive populations (originated from the Ta-pa Mountains (Tp), the midrange of the Qinling Mountains (Ql) and the Western Sichuan Plateau (WS), respectively) to evaluate the genetic variations. A total of more than 3.15 billion high-quality clean reads and 4.37 million high-quality SNPs were generated by RAD sequencing. Based on the analysis, we found that captive forest musk deer populations exhibit a relatively low level of genetic diversity. Ql displayed a higher level of genetic diversity than the Tp and WS populations. Tp and WS had experienced population bottlenecks in the past as inferred from the values of Tajima’s D. There were high levels of heterozygote deficiency caused by inbreeding within the three populations. Population structure analysis suggested that the three populations have evolved independently, and a moderate amount of genetic differentiation has developed, although there was a low level of gene flow between the Ql and Tp populations. Furthermore, the average quantities of musk secreted by musk deer in the Tp and WS populations were significantly higher than that in the Ql population. The present genetic information should be considered in management plans for the conservation and utilization of musk deer from captive breeding.

There are six musk deer (Moschidae) species in the world (Jiang *et al.* 2015), and all are famous for the musk secreted by the male gland, a precious traditional Chinese medicine and a superior component in perfume production (National Pharmacopoeia Committee 2015). However, due to over-exploitation and habitat fragmentation, the wild musk deer populations plummeted from 2.5 million in the 1950s to 66,300 in the year 2000 (Sheng and Liu 2007; Ma and Zhang 2009), and each species has been listed on the Category I of the State Key Protected Wildlife List of China. Artificial breeding is an effective way to protect musk deer, in that it is beneficial to population growth and that it can mitigate the poaching pressure on wild populations. With the support of the Chinese government, several communal musk deer farms were established in 1958, and the founder animals were caught from the nearby mountains. To date, the farmed species include forest musk deer (*Moschus berezovskii*) (FM Deer), alpine musk deer (*Moschus chrysogaster*) (AM Deer) and Siberian musk deer (*Moschus moschiferus*) (SM Deer). Among these, the FM Deer is the main species used in breeding programs. Whereas, the production from captive populations remained constant over the subsequent four decades until the beginning of the present century (Sheng and Liu 2007). Since then, significant success has been achieved in the artificial propagation of FM Deer, and the number reached 20,000 individuals in 2017, of which 90% were distributed in Shaanxi and Sichuan Provinces.

Musk deer breeders have accumulated a rich body of experience in all aspects of breeding management (Cheng and Zou 1991; Huang *et al.* 1998), disease prevention (Wang *et al.* 2007; Li *et al.* 2014) and the regulation of musk secretion (Bi *et al.* 1980; Hong *et al.* 1981) during FM Deer breeding. Nonetheless, several problems have become more prominent with the population growth, such as high incidence of disease and low offspring survival rates, especially in populations that exceed one hundred individuals. This may be related to the population degradation caused by founder effects and inbreeding (Ding *et al.* 2009), implying the need to increase and conserve genetic resources. The key to genetic conservation is maintaining genetic diversity, which is essential for preserving the evolutionary potential in response to environmental changes. In addition, investigating genetic diversity can provide valid information for improvement of FM Deer breeding (Frankham *et al.* 2002).

Previous studies have assessed the genetic diversity of FM Deer by using a variety of molecular markers, such as amplified fragment length polymorphism (AFLP) (Zhao *et al.* 2011), mitochondria DNA (mt DNA) (Peng *et al.* 2009; Feng *et al.* 2016), microsatellite (Guan *et al.* 2009; Zhao *et al.* 2011; Huang *et al.* 2013) and major histocompatibility complex (MHC) genes (Yao *et al.* 2015; Xia *et al.* 2016). These studies indicated that a risk of diversity loss existed in domestic FM Deer. However, the existing genetic variability and population structure of FM Deer were not sufficiently researched due to the small sample sizes, the limited number of markers employed, and the use of populations from the same geographical area. Compared to the above genetic markers, single nucleotide polymorphisms (SNPs) are ideal markers because of their dense distribution across the genome, their genetic stability, and ease of detection (Marnellos 2003). SNPs have been widely used in population genetics analysis and evolutionary studies in recent years following the development of high-throughput sequencing techniques (Nielsen *et al.* 2011). Restriction-site associated DNA sequencing (RAD-seq) can generate a large number of SNPs at a simplified genome-wide scale that can provide sufficient information to genetic research (Davey *et al.* 2011).

In our study, a total of 274 FM Deer samples collected from three farms and used to estimate the level of genetic diversity and population structure using RAD sequencing. The three farmed populations originated from different regions that together nearly cover the areas of origin of the captive FM Deer. In addition, we evaluated whether there were differences in musk secretion among the three populations. The results of this study provide informative data for the conservation and utilization of musk deer, and the results should be helpful for breeders attempting to develop a healthy genetic breeding strategy.

## Materials and Methods

### Study area and sample collection

**Figure 1:**
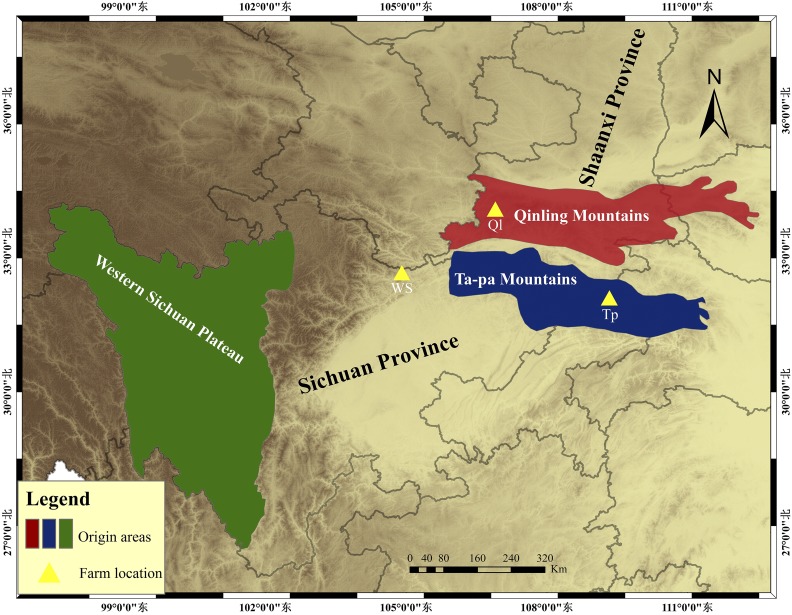
Sampling farm localities and origin areas. The forest musk deer samples were collected from the Tp farm, Shaanxi Province; the Ql farm, Shaanxi Province; and the WS farm, Sichuan Province. Forest musk deer from Tp, Ql, and WS farms were originally from the Ta-pa Mountains, the midrange of the Qinling Mountains and the Western Sichuan Plateau, respectively.

Ear tissue samples of 274 FM Deer were obtained from three captive farms from one company, with 59 individuals from the Tp farm located in the southernmost area of Shaanxi, 192 individuals from the Ql farm located in the southwest of Shaanxi and north of the Qinling Mountains, and 23 individuals from the WS farm located in the north of Sichuan province (Figure 1; Table 1). FM Deer from Tp, Ql and WS farms were originally from the Ta-pa Mountains, the midrange of the Qinling Mountains and the Western Sichuan Plateau, respectively (Figure 1; Table 1). These areas are the original habitats of the captive FM Deer populations, and these farms are typical representatives of the musk deer artificial breeding industry in China.

**Table 1:** Information of sample collected

Samples and sources	Sample number	Musk deer captive farm location		Musk deer’s origin
Ear tissue samples from forest musk deer	59	Tp farm: southernmost area of Shaanxi		Ta-pa Mountains
	192	Ql farm: southwest of Shaanxi and north of the Qinling Mountains		Qinling Mountains
	23	WS farm: north of Sichuan province		Western Sichuan Plateau
Alpine musk deer	6	Tp farm	Farm location are same as above	Xinglong Mountains
	6	Ql farm		
	16	WS farm		

The three farms are independent and do not exchange musk deer with each other. The 274 individuals were all adult males aged 2.5-6.5 years and were selected randomly from productive groups in each farm. FM Deer from the same farm had similar feeding conditions. Samples collection was under the permit from the Forestry Department and conformed to the National Wildlife Conservation Law in China.

Meanwhile, 28 AM Deer for which there were records of musk secretion were selected randomly from the above farms, with 6, 6 and 16 individuals from the Tp, Ql and 16 WS farms, respectively (Table 1). All of the AM Deer were originally from the Xinglong Mountains in Gansu province and were all introduced into the three farms after the year 2000. These AM Deer were used as a reference standard in the comparative analysis of musk secretion.

### DNA isolation, RAD library construction and sequencing

DNA was isolated from ear tissue samples using the traditional method of SDS and proteinase K referred to in Sambrook *et al.* (1989). DNA purity, density and integrity were estimated by NanoDrop, Qubit and 1% agarose gel electrophoresis, respectively.

RAD-seq libraries were constructed using a protocol adapted from Baird *et al.* (2008). Briefly, genomic DNA from each individual was digested separately with *Eco*R I and then heat inactivated at 65°. Various P1 adapters, each with a unique 4-8 bp molecular identifying sequence (MID), were then ligated to designated individuals, which were then pooled in groups and randomly sheared to DNA fragments. Sheared DNA was purified, eluted, and separated using gel electrophoresis, and a DNA fraction corresponding to 300-700 bp was excised and purified. After end repair, purification, and elution, dATP overhangs were added to the DNA fraction. A paired-end P2 adapter containing T overhangs was ligated to the sheared, size-selected, P1-ligated, and pooled DNA template with a specific adapter. The ligated material was then purified, eluted, and subjected to PCR enrichment. RAD products were sequenced using 150 bp pair ends and the data for each individual were extracted according to the specific MID.

All libraries underwent high-throughput sequencing using an Illumina HiSeq 2500 platform at the Gene-denove Bioinformatics Institute (Guangzhou, China).

### Clean reads filtering

Quality trimming is an essential step to generate high confidence of variant calling. Raw reads would be processed to get high quality clean reads according to three stringent filtering standards:

1. Removing reads with ≥ 10% unidentified nucleotides (N);
2. Removing reads with > 50% bases having phred quality scores of ≤ 20;
3. Removing reads aligned to the barcode adapter.

### De novo assembly of RAD tags

We would filter out Illumina short reads lacking sample-specific MIDs and expected restriction enzyme motifs before reads clustering. All the short reads from each of the samples were then clustered into stacks by the stack program (http://catchenlab.life.illinois.edu/stacks/) on the basis of sequence similarity of the first read (allowing three mismatches at most between any two reads within each tag read cluster, with clusters having < 3 discarded) (Catchen *et al.* 2011; Willing *et al.* 2011). The paired-end reads associated with each RAD cluster tag were extracted to construct scaffolds using adjacent contigs identified by paired-end information.

As the musk deer lacked genome data, RAD tags served as a reference sequence for subsequent mutation detection and advanced analysis.

### SNP and InDel identification

To identify SNP and insertions and deletions (InDel), the Burrows-Wheeler Aligner (BWA) was used to align the clean reads from each sample against the RAD tags with the settings ‘mem 4-k 32 -M’, -k is the minimum seed length, and -M is an option used to mark shorter split alignment hits as secondary alignments (Li and Durbin 2009). Variant calling was performed for all samples using the GATK’s Unified Genotyper. SNPs and InDels were filtered using GATK’s Variant Filtration with proper standards (-Window 4, -filter “QD < 2.0 || FS > 60.0 || MQ < 40.0”, -G_filter “GQ < 20”).

### Estimates of population genetic diversity

All of the above filtered SNPs were used to calculate θ_π_ (the nucleotide diversity), θ_ω_ (Watterson’s estimator) and F_ST_ (population differentiation) using an approach described by Fumagalli *et al.* (2013; 2014). Tajima’s D values were also estimated to detect the selection effect as described by Korneliussen *et al.* (2013). Only SNPs with a missing rate < 0.2 and minor allele frequency (MAF) ≥ 0.05 were used to calculated F_IS_ (inbreeding coefficient of an individual relative to the subpopulation) and F_IT_ (overall inbreeding coefficient of an individual relative to the total population) according to Nei (1983). Deviation from Hardy-Weinberg equilibrium (HWE) was assessed using the PLINK (version 1.9) software (Chang *et al.* 2015).

### Population structure analysis

All of the filtered SNPs were used to carry out genetic structure analyses. To estimate relationships, the distance matrices were calculated among all individuals using PHYLIP 3.69 (Plotree and Plotgram 1989). A neighbor-joining tree was constructed and displayed in MEGA 4.0, and a total of 1,000 replicates were used to generate bootstrap values. A principal component analysis (PCA) were performed to preliminarily classify the population subdivision pattern in the software GCTA (Yang *et al.* 2011). Population genetic structure was further inferred using the admixture model in the ADMIXTURE software (version 1.3.0), and the parameters used defaults settings (Alexander *et al.* 2009). The pre-defined genetic clusters (K) was set from 2 to 5.

### Statistical analysis

The data for an individual’s quantity of musk secretion were recorded during at least two years, and unqualified musk (judged by color and odor) was recorded as “0 g.” The individual’s annual average quantity of musk was used for the comparative analysis of musk secretion among Tp, Ql and WS farms. The comparison didn’t include individuals with an average musk yield of 0 g. Kolmogorov-Smirnov tests were performed to verify the distribution of musk secretion quantity data for each population. When the data showed a normal distribution, one-way ANOVA was used to test for differences in the quantity of musk secretion among the three farms, and the Kruskal-Wallis H test was used when the data deviated from a normal distribution. All of the data were presented as mean ± SD. All of the statistical tests were carried out using SPSS 20.0 software, and the significance level was set to 0.05.

### Data availability

All raw sequences of the 274 FM Deer have been deposited in the NCBI Short Read Archive with project accession: SRP161974. Supplementary information have been upload to the figshare (https://figshare.com/s/5a9129a307d00c94f61d). Figure S1 and Table S1 contain detailed SNP and filtered SNP statistics. Figure S2 and Table S2 contain detailed InDel statistics. Table S3 contains statistics information of re-divided groups. Supplemental material available at Figshare: https://figshare.com/s/5a9129a307d00c94f61d.

## Results

### RAD sequencing

Illumina sequencing of 274 FM Deer generated a total of 3,201,312,984 raw reads, of which 3,155,404,082 (98.57%) high quality clean reads were generated by quality trim. A total of 188,761,630 stacks were yielded by cluster of clean reads basis of sample-specific MID, in which the average number of stacks per individual was 688,911, with amounts ranging from 148,853 to 1,369,592. The average depth achieved was 5.32 × per individual, varying from 4.196 × to 7.362 ×. In addition, FM Deer’s *de novo* assembly of the paired-end reads yielded 2,587,808 RAD tags, in which the average length of RAD tags was 250 bp, changing from 100 bp to 4043 bp.

### SNP and InDel calling

SNP calling was performed from 274 samples, and 4,371,985 high-quality SNPs were identified, with nearly twice the number of transitions (2,891,633) than transversions (1,480,352) (Table 2; Figure S1A). The SNPs were then pooled into three groups, with 4,138,277 from Tp, 4,336,478 from Ql and 4,011,444 from WS. A large proportion of SNPs (3,887,961 of 4,371,985 or 88.93%) were shared among the three populations, indicating that there is substantial similarity in genetic background among the three populations (Figure S1B).

**Table 2:** Summary of SNP statistics in forest musk deer from three farms

Population	SNP types and number			θ_π_	θ_ω_	Tajima’s D
	Total	Transition	Transversion			
Tp	4,138,277	2,745,125	1,393,152	6.32 × 10^−4^	5.94 × 10^−4^	3.61 × 10^−6^
Ql	4,336,478	2,869,122	1,467,356	9.00 × 10^−4^	1.04 × 10^−3^	−3.91 × 10^−5^
WS	4,011,444	2,665,826	1,345,618	5.91 × 10^−4^	5.37 × 10^−4^	3.32 × 10^−5^
Total	4,371,985	2,891,633	1,480,352	1.10 × 10^−3^	1.42 × 10^−3^	−2.29 × 10^−5^

There were a total of 211,998 InDels identified, with 134,483 from Tp, 198,967 from Ql and 117,573 from WS (Table S1). Insertions and deletions ranged from 1-44 bp and 1-69 bp in length, respectively, of which a substantial proportion (194,298 of 211,998, or 91.65%) of the InDels were relatively small (1-6 bp), with only 2.14% (or 4,545) greater than 20 bp in length (Figure S2).

The numbers of SNPs and InDels in Ql were higher than those in the Tp and WS populations, indicating that Ql had the highest level of genetic variation. The lower levels of diversity in Tp and WS were detected using θ_π_ and θ_ω_, and the values were lower compared with that of the Ql population (Table 2). Tajima’s D statistics were positive for the Tp and WS populations, supporting the occurrence of recent population bottlenecks in the two populations (Table 2).

A total of 256,953 SNPs were generated according to the missing rate < 0.2 and MAF ≥ 0.05 (Table S2). The SNPs were then pooled into three populations, with 241,411 from Tp, 255,774 from Ql and 245,889 from WS. Of these SNPs, a high proportion deviated from HWE in each population (*P* < 0.01). Positive values of F_IS_ were also found in the three populations, with values ranging from 0.34 (WS) to 0.56 (Ql) (Table S2).

### Population structure analysis

**Figure 2:**
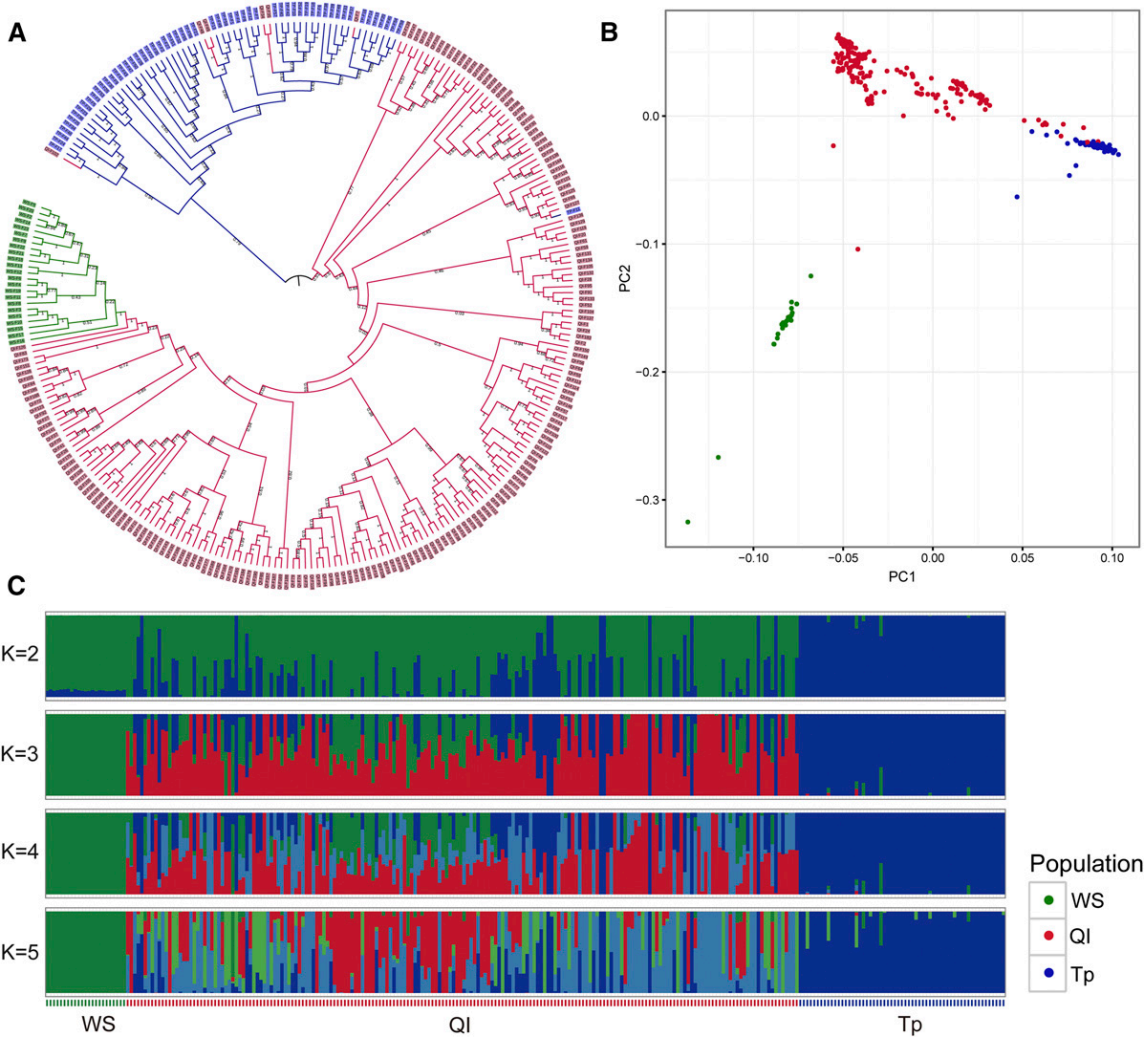
Phylogenetic relationships and population genetic structure. (A) A neighbor-joining tree for the forest musk deer constructed by PHYLIP (version 3.69) based on the high-quality SNPs. The numbers in the figure represent bootstrap values. (B) Principal component analysis (PCA) of forest musk deer was implemented in GCTA. The plot was based on the first two principal components. (C) Population genetic structure was inferred by Admixture (version 1.3.0). The number of populations (K) was pre-defined from 2 to 5 and each color represents one putative ancestry background. The Y axis quantifies group membership and the X axis shows different individuals.

To explore the genetic relationships among the three populations, a neighbor-joining tree was constructed based on the SNP data. The FM Deer clustered into two branches in which Tp and Ql as sister lineages evolved independently, while WS was differentiated from the Ql population and formed a separate group (Figure 2A). Some individuals from Ql clustered together with the Tp population, and one Tp individual clustered with the Ql population, providing evidence of possible gene flow between the Tp and Ql populations (Figure 2A). To estimate the level of genetic differentiation, F_ST_ values were calculated, and the value between the Ql and WS populations was lower (0.065) than the others (Tp *vs.* Ql: 0.081 and Tp *vs.* WS: 0.088), corresponding to the relationship shown above. The PCA revealed a similar result, dividing the individuals into three groups. The WS population was clearly separated from the other populations in the first two PCs, while the Ql population was sandwiched between the other populations and overlapped with the Tp population, suggesting a higher similarity of genetic background between Ql and the others (Figure 2B). These results were further confirmed using an admixture model. When K was set to 2, Tp was clearly separated from the Ql and WS populations, and the latter two populations were further separated when K was set to 3, suggesting a closer relationship between these two groups than either population with the Tp population. When K was set at 4 or 5, most individuals in Ql had two or three lineages, whereas the lineages for WS were always single, confirming that WS was a descendant of a closed population without gene flow from other populations (Figure 2C).

### Comparison of the quantity of musk secretion among the three farms

According to the recorded musk data, there were large individual differences in musk secretion among the 274 FM Deer. There were 15 individuals that produced more than 20 g of musk, with amounts ranging from 20 g to 24.3 g. In contrast, the average musk weight of 37 individuals was less than 5 g, with 11 Tp, 22 Ql and 4 WS individuals. There were 20 individuals that secreted 0 g or unqualified musk during 2-3 consecutive years, covering 8, 10 and 2 individuals from the Tp, Ql and WS populations, respectively. The Tp (13.56%) and WS (8.7%) populations had a higher proportion of individuals that secreted 0 g or unqualified musk compared with the Ql population (5.2%).

**Figure 3:**
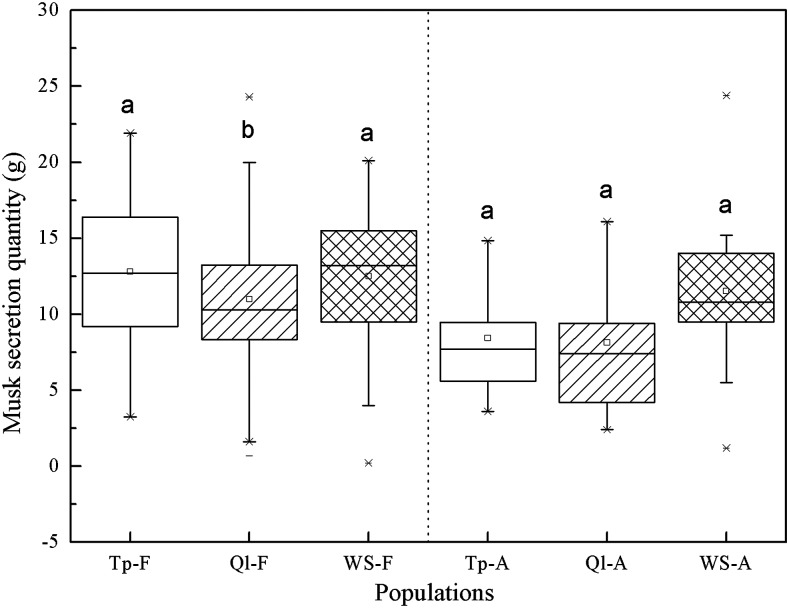
Comparison of the average amount of musk secretion among three farms. Populations Tp-F, Ql-F and WS-F represent forest musk deer from Tp, Ql and WS farms, respectively. Populations Tp-A, Ql-A and WS-A represent alpine musk deer from Tp, Ql and WS farms, respectively. Different letters indicate significant differences at the *P* < 0.05 level.

The average quantities of musk for the FM Deer populations were as follows: Tp-F (12.82 ± 4.76 g) > WS-F (12.51 ± 4.79 g) > Ql-F (10.99 ± 4.79 g), and the amount in both Tp and WS populations was significantly higher than in the Ql population (Tp *vs.* Ql, *P* = 0.013; WS *vs.* Ql, *P* = 0.047; Tp *vs.* WS, *P* = 0.806) (Figure 3). To further eliminate the impact of management differences, we conducted a comparative analysis among AM Deer populations from the above three farms. The musk weights of the AM Deer populations were as follows: WS-A (11.52 ± 5.10 g) > Tp-A (8.43 ± 3.87 g) > Ql-A (8.13 ± 4.80 g), while there were no significant differences (*P* > 0.05) among these populations (Figure 3).

## Discussion

The musk deer captive breeding industry is thriving in China, and many private farms have been built during the past decade. Nationally, western China has long been the main area for the musk deer breeding industry, especially the Shaanxi and Sichuan Provinces. In our study, three typical captive populations from the two above provinces were selected to evaluate their genetic structure. The present research could provide effective guidance for FM Deer population management.

### Reasons for differences in genetic diversity among the three populations

The level of genetic diversity is closely related to a population’s viability, meaning that a population with a higher level of genetic diversity has greater adaptability, conducive to its long-term survival (Frankham *et al.* 2002; England *et al.* 2003, Reed and Frankham 2003). The population genetic diversity is affected by a number of complex demographic factors, including population changes, age structure and migration (Beebee and Rowe 2008). In our study, the Ql population showed the highest level of genetic diversity, which could be explained by the historical breeding information. First, the Ql population employed the largest number of musk deer as founders among the three farms. Previous studies also confirmed that population genetic diversity was affected by the number of founder animals, and a larger number of founders would reduce the decline of genetic diversity (Broders *et al.* 1999; Williams *et al.* 2002; Zhang *et al.* 2007). Second, the Ql farm was structured as open range, which allowed wild individuals to be introduced intermittently over the past years; some individuals were always exchanged with other surrounding farms since the foundation, whereas the other two populations were relatively closed. The introduction of animals would incorporate new alleles and allow genetic improvement in the breeds (Guan *et al.* 2009). Combined with the results of a previous study by Feng *et al.* (2014), we consider that the Ql population could be used as a valuable gene pool for genetic improvement in captive breeding. Meanwhile, the occurrence of a population bottleneck also can lead to a reduction of population genetic diversity (Beebee and Rowe 2008). The Tp and WS populations had experienced population bottlenecks, as inferred by the positive values of Tajima’s D. These bottlenecks may have been caused by the dramatic decline of effective population sizes in the past. Tp and WS had smaller numbers of founders than recommended at the inception of the farms in 1958 (Frankham *et al.* 2002). Furthermore, the population sizes have not grown significantly due to mismanagement that led to deaths of musk deer, although wild individuals were introduced continuously during the next several years after the foundation.

### Relatively low levels of genetic diversity in captive FM Deer populations

According to the filter criteria of Ba *et al.* (2017), the average frequency of heterozygous SNPs per kilobase pair is 0.54 in the genome of captive FM Deer individuals. This value was lower than that in captive sika deer (0.74) (Ba *et al.* 2017), breeding cattle (1.35) (Bovine HapMap Consortium *et al.* 2009) and giant panda (1.32) (Zhao *et al.* 2013), but was very similar to that of Milu deer (0.51) (Zhu *et al.* 2016), a species that nearly went extinct during the Han Dynasty and experienced severe genetic drift. This suggests that there was a relatively low level of genetic diversity in the captive FM Deer populations. In addition to the breeding domestication, a low amount of genetic diversity in the wild populations before creation of the farms may be one of the reasons for this phenomenon, considering that the time in captivity has been relatively short and that wild individuals have been introduced into the populations.

In our study, all populations significantly deviated from HWE at most SNP loci (*P* < 0.01). Admittedly, this may have been affected by the genotyping accuracy, but in our research the large sample size could effectively remedy the genotype error caused by the relatively low sequencing depth. Many other factors could have caused the deviation from HWE, including inbreeding, genetic drift, Wahlund effects (presence of population substructure) and natural selection (Nei and Kumar 2000). All of the deviations were related to the positive value of F_IS_ observed in our research, indicating that the deviation toward a direction of heterozygote deficit and inbreeding may be the main reason for the high levels of deviation from HWE (Table S2). Additionally, the higher levels of deviation from HWE in the Ql and Tp populations may be explained by Wahlund effects (Figure 2), meaning that the presence of substructure has resulted in a lack of population heterozygosity.

The value of F_IS_ observed in our study was obviously higher than the values reported in other captive populations of cervidae, for example, in red deer (0.23±0.13) (Kasarda *et al.* 2017), sika deer (0.16) (Ba *et al.* 2017) and hog deer (-0.0302 ± 0.0062) (Wang *et al.* 2017). This means that inbreeding has caused a high level of heterozygote deficiency in captive FM Deer. In the production practice, only males with superior traits participate in mating, which suggests that directional breeding could lead to nonrandom mating and consequently to genetic drift. These results supported those of previous studies concluding that inbreeding and genetic drift were the main reasons for the decline of genetic diversity in captive populations (Guan *et al.* 2009; Ba *et al.* 2017), and that there was a risk of diversity loss in domestic FM Deer. To our knowledge, inbreeding may have led to depression of traits related to fitness (Hedrick and Kalinowski 2000). In our study farms, some individuals in the Tp and WS populations appear to have been experiencing inbreeding depression, as reflected in weakened disease resistance and an increase in neonatal morbidity.

### Evolutionary history and genetic differentiation of domestic FM Deer

Optimizing management measures also requires a thorough understanding of the population evolutionary history and genetic differentiation (Bergl and Vigilant 2007). Our study showed that the WS was differentiated from Ql and was closely related to the Ql population. Together with the PCA results, these findings prompted us propose that the population in the Western Sichuan Plateau was descended from the population in Qinling Mountains, and subsequently suffered from selection and became isolated. In general, F_ST_ values ranging from 0.05 to 0.15 indicates a medium level of differentiation between populations (Wright 1990). In this study, the value of F_ST_ varied from 0.065 (between WS and Ql populations) to 0.088 (between WS and Tp populations). This suggests that a medium level of differentiation between these populations, and that this was likely to be partly shaped by historical factors and not entirely caused by genetic drift with captive breeding. Geographic isolation was one possible reason for the genetic differentiation among the three populations (Yang *et al.* 2003). Admittedly, the time span of the isolation was not very long in wild FM Deer considering the large population size and extensive distribution of the species throughout its history; therefore the population differentiation appears to be at a medium level. Evidence for gene flow only exists between the Tp and Ql populations, demonstrating that those populations may be not have been completely isolated between Qinling and the Ta-pa mountains in the past. Meanwhile, the result also suggests that the population from the western Sichuan plateau may be thoroughly isolated from others. Reduction or termination of gene flow caused by habitat fragmentation can give rise to a loss of genetic diversity in wild populations (Frankham *et al.* 2002). Thus, the lower diversity of the original population may be one of the secondary reasons for the lowest genetic diversity being observed in the WS population.

### Effects of artificial breeding and nutrition on musk secretion

In addition to maintaining genetic health, it is important to increase the musk yield in captive farms. Directional selection will lead to an improvement of production performance (Zhang 2001). In our study, the average amount of musk secretion in the Tp and WS populations was significantly higher than that in Ql population (*P* < 0.05). The Tp and WS farms were established 30 years earlier than the Ql farm, and the longer period of directional selection may have generated more homozygous genes contributed to musk secretion, resulting in an increase of musk production. Furthermore, differences in nutrition levels, especially in crude protein, also affect the quantity of musk secretion (Cheng *et al.* 2002). At the Ql farm, there was a lack of fresh leaves in winter, and pumpkins and carrots were used as supplementary food for the FM Deer. Thus, lower levels of crude protein intake may be another reason for the low musk yield of the Ql population; this was further confirmed by the comparison of musk secretion with the AM Deer populations. This implies that protein should be replenished in musk deer farms located in the north of the Qinling Mountains.

In our research, we also found that the Tp and WS populations had higher proportions of individuals whose average musk yield was 0 g than in the Ql population, which may be related to the depression caused by inbreeding and other factors. Following this result, we re-divided the musk deer individuals into four groups according to the amount of musk secretion (Table S3). We found that the P group (including individuals whose musk secretion was 0 g or unqualified) had the lowest level of genetic diversity. Furthermore, the values of Tajima’s D in the L group (including individuals who secreted low amounts of musk: 1 g-7 g) and the P group were significantly higher than those in other groups, which may be associated with the accumulation of low-frequency detrimental mutations in these groups. A population with lower genetic diversity and a higher detrimental mutation tends to be more susceptible to pathogens, and the resulting disorders may be the best explanation for the phenomenon (Xia *et al.* 2016).

### Suggestion for breeding management

The present research into genetic diversity and population structure can provide valid information for FM Deer management and conservation. Indeed, we found high levels of heterozygote deficit and significant genetic structure in the three FM Deer populations. To avoid genetic degradation, we offer the following suggestions: (1) Inbreeding should be avoided to some extent, and individuals displaying traits of inbreeding depression should be weeded out immediately. The kinship of all individuals (data unreleased) identified in our studies could be useful for avoiding inbreeding. (2) The three populations should be treated as distinct units. Genetic evaluation is necessary before introduction, and we advise against optional introductions between these regional populations. (3) In practice, nutrition levels, especially for crude protein, should be supplemented in the farms. Managers should balance the genetic diversity and musk production, and establishing a preserved population and a breeding population may be a feasible way to relieve the conflict.

## Conclusion

In summary, this research has provided the first description of genetic variability and differentiation in three forest musk deer populations that originated from different geographic areas. Our results reflect the genetic status of the musk deer industry in China. There was a relatively low level of diversity in captive forest musk deer and a medium level of genetic differentiation had developed between captive populations. Populations in the Qinling Mountains could be used as a valuable gene pool in genetic improvement. These results provide insight into genetic improvement and conservation for the musk deer.
